# Exercise Right Ventricular‐Pulmonary Arterial Coupling and Functional Outcome in Patients Undergoing Surgery for Secondary Ischemic Mitral Regurgitation

**DOI:** 10.1161/JAHA.124.037198

**Published:** 2025-04-11

**Authors:** Carlo Fino, Diego Bellavia, Michele D'Alonzo, Maurizio Merlo, Vito D. Bruno, Julien Magne, Massimo Caputo, Amedeo Terzi, Michele Senni, Samuele Bichi, Caterina Simon, Edoardo Sciatti, Giovanni Marchetto, Attilio Iacovoni, Philippe Pibarot

**Affiliations:** ^1^ Cardiovascular and Transplant Department Ospedale Papa Giovanni XXIII Bergamo Italy; ^2^ Division of Cardiovascular Diseases Mediterranean Institute for Transplantation and Advanced Specialized Therapies (ISMETT) Palermo Italy; ^3^ Cardiac Surgery Department Henri‐Mondor University Hospital Creteil France; ^4^ University of Bristol Medical School – Translational Health Science Bristol UK; ^5^ Service de Cardiologie CHU Limoges Limoges France; ^6^ Fondazione IRCCS San Gerardo dei Tintori Monza Italy; ^7^ Quebec Heart and Lung Institute Quebec Canada

**Keywords:** 6‐minute walking test, exercise stress echocardiography, right ventricular – pulmonary artery coupling, secondary ischemic mitral regurgitation, Cardiovascular Surgery, Echocardiography, Exercise Testing, Heart Failure, Valvular Heart Disease

## Abstract

**Background:**

The exercise assessment of the right ventricular‐pulmonary arterial (PA) coupling adds diagnostic and prognostic value in patients with heart failure. In patients with ischemic mitral regurgitation undergoing surgery, data on the exercise assessment of the right ventricular‐PA coupling are not available. Resting and exercise echocardiographic predictors of functional outcome in patients with ischemic mitral regurgitation were tested.

**Methods:**

Six‐minute walking test and exercise echocarrdiogram performed at baseline, at 1 years, and at a median follow‐up of 6 years (interquartile range, 3.70; range, 4.5–8) on 50 patients (67±8 years; ejection fraction: 35±5%) undergoing valve replacement or repair. Linear mixed models were used to evaluate the predictive value of preoperative echocardiographic parameters on the longitudinal distribution of the 6‐minute walking test.

**Results:**

Preoperative exercise tricuspid annular plane systolic excursion (TAPSE)/PA systolic pressure strongly correlated with the long‐term 6‐minute walking test (r=0.81, *P*<0.01). The receiver operating characteristic analysis found a preoperative exercise TAPSE/PA systolic pressure <0.34 predicted the lowest quartile of the 6‐minute walking test in the long term (sensitivity: 79%; specificity: 100%) as well as a composite outcome of heart failure and death from any cause (positive predictive value: 91.3%, negative predictive value: 100%). On multivariable analysis, TAPSE and TAPSE/PA systolic pressure were significantly associated with a better long‐term 6‐minute walking test.

**Conclusions:**

A preoperative exercise TAPSE/PA systolic pressure <0.34 predicts a poor functional performance and a higher likelihood of clinical adverse events. In patients with ischemic mitral regurgitation the exercise right ventricular ‐PA coupling could improve risk stratification. Larger studies are needed.

**Nonstandard Abbreviations and Acronyms**
6‐MWT: 6‐minute walking test
ESE: exercise stress echocardiography
MVR: mitral valve replacement
PASP: pulmonary artery systolic pressure
RMA: restrictive mitral annuloplasty
SIMR: secondary ischemic mitral regurgitation
TAPSE: tricuspid annular plane systolic excursion

**Clinical Perspective**

**What Is New?**

- In patients with secondary ischemic mitral regurgitation undergoing surgery, a preoperative exercise tricuspid annular plane systolic excursion/pulmonary artery (PA) systolic pressure ratio is an independent predictor of functional capacity at long term. A tricuspid annular plane systolic excursion/PA systolic pressure <0.34 predicts the lowest quartile of 6‐minute walking test and a composite outcome of heart failure and death for any cause.

**What Are the Clinical Implications?**

- The exercise tricuspid annular plane systolic excursion/PA systolic pressure assessment of patients with ischemic mitral regurgitation may unmask those who exhibit a latent resting RV‐PA uncoupling before the occurrence of RV dilation, dysfunction, and failure.
- Evaluation of RV‐PA coupling may be useful to enhance patient risk stratification, to guide the patient‐specific selection process for the type of intervention (surgical versus nonsurgical intervention), to assess and monitor the efficacy of the procedure.

In patients with secondary ischemic mitral regurgitation (SIMR) the prognosis remains dismal.^1^, ^2^ In such challenging patients an abnormal ventricular‐arterial coupling plays a role in the impairment of the cardiac reserve with a functional capacity reduction, leading to a poor quality of life and increased mortality.^3^ In this respect, assessing the right ventricular (RV)‐pulmonary arterial (PA) coupling during exercise, add a significant diagnostic and prognostic value.^4^, ^5^, ^6^, ^7^, ^8^, ^9^, ^10^, ^11^ The 6‐minute walking test (6‐MWT) represents an objective quantitative method to evaluate functional capacity^12^ and its change during follow‐up provides data on the prognosis and treatment efficacy.^3^, ^13^, ^14^ Exercise stress echocardiography (ESE) contributes to guide the clinical management and to assess the durability and the impact of valvular interventions on functional recovery.^3^, ^15^, ^16^ To date no studies have investigated the predictive value of exercise RV‐PA coupling on long‐term functional capacity in patients with SIMR undergoing surgery.

The objective of this study was therefore to test resting and ESE predictors of early and long‐term functional capacity in patients undergoing surgery for severe SIMR.

## METHODS

The data that support the findings of this study will be available on reasonable request from the corresponding author.

For this study, the Strengthening the Reporting of Observational Studies in Epidemiology cohort reporting guidelines were used.^17^

### Population

From 2009 to 2019 137 consecutive patients with SIMR underwent restrictive mitral annuloplasty (RMA) or mitral valve replacement (MVR) and coronary artery bypass graft, at Papa Giovanni XXIII Hospital, Bergamo, Italy. Definition of SIMR was (1) MR >1 week after myocardial infarction; (2) at least 1 of left ventricular segmental wall motion abnormality; (3) significant coronary artery disease (≥75% stenosis of ≥1 coronary vessel); (4) structurally normal MV; and (5) type IIIb MR according to Carpentier classification, with or without annular dilatation.

**Figure 1:**
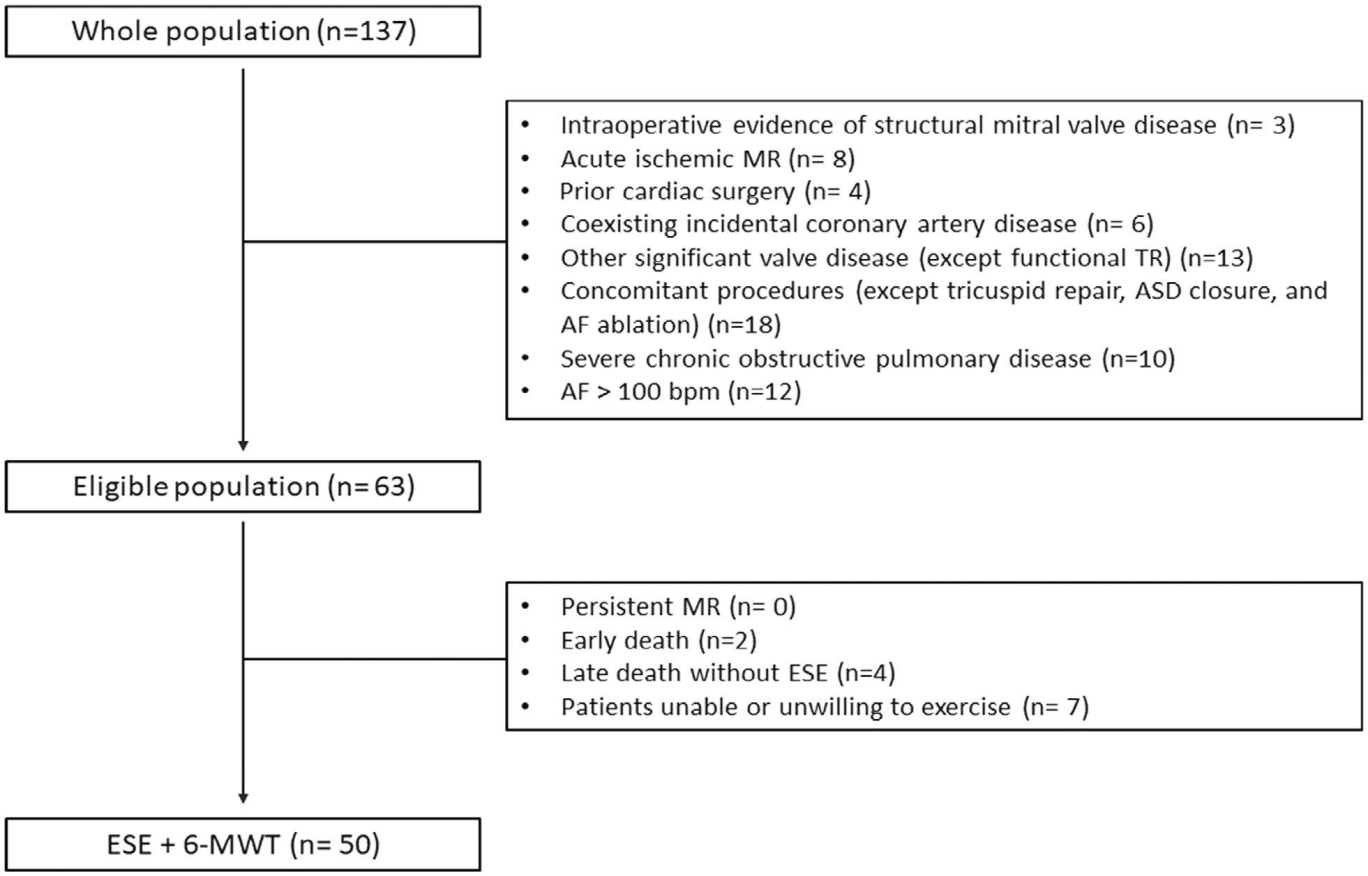
Flow chart describing the patient population. Persistent MR defined by postoperative VC >3 mm, before discharge. 6‐MWT indicates 6‐minute walking test, expressed in meters; AF, atrial fibrillation; ASD, atrial septal defect; ESE, exercise stress echocardiography; MR, mitral regurgitation; and TR, tricuspid regurgitation.

Surgical indication was assessed during multidisciplinary meeting. During the first period of this study, given the lack of evidence of superiority of RMA versus MVR, the selection of the technique was left to the surgeon's discretion. Afterwards according to recent evidence, MVR was favored.^18^, ^19^, ^20^ A complete list of exclusion criteria is shown in Figure 1. The final study population was composed of 50 patients (RMA, n=20; MVR, n=30) on whom we performed a 6‐MWT and ESE at preoperative baseline, at 1 year and at last follow‐up (FU) (Figure 1).

ESE was performed by cardiologists, blind to clinical information and according to the American Society of Echocardiography recommendations.^21^ The ESE protocol has been previously described.^22^ On the same day of the ESE, a physician, blind to the study as well, performed the 6‐MWT according to American Thoracic Society guidelines.^23^ All patients received the same perioperative and postoperative care. Operative, imaging, and clinical data were prospectively collected in our institutional database. FU was completed in all patients.

The study complied with the Declaration of Helsinki and was approved by the institutional review board (ASST Papa Giovanni XXIII; registration number: 2022‐0056). Written informed consent was obtained from all patients. All authors had full access to all study data and take responsibility for their integrity and the data analysis.

### Surgery

All procedures were performed by median sternotomy with bicaval cannulation and systemic cooling at 32 °C. A conventional left atriotomy was used. Myocardial protection was achieved with intermittent antegrade and retrograde warm blood cardioplegia. Visual inspection by the surgeon confirmed the absence of any structural abnormalities. Patients received a complete annuloplasty ring (Carpentier‐Edwards Physio ring, Edwards Lifesciences, Irvine, CA) undersized by 2 sizes. The ring was selected by measuring the intercommissural distance of the MV and positioned to cover the surface of the stretched middle scallop of the anterior leaflet.^24^ For patients undergoing valve replacement, all the prostheses were inserted with systematic preservation of subvalvular apparatus. The choice between biological versus mechanical prosthesis was a shared decision between surgeon and patient, based on age, life expectancy, preference, and indication/contraindications for warfarin therapy.^25^

A complete revascularization was defined as placement of at least 1 graft distal to an approximately 50% diameter narrowing in each of the 3 major vascular systems, when this stenosis intraoperatively corresponded to a vessel with a diameter of ≥1.5 mm.^26^ According to this definition, we achieved complete revascularization in all patients.

Intraoperatively transesophageal echocardiography was performed for assessing residual MR and to rule out periprosthetic leak. Exclusion of residual persistent MR (vena contracta ≥3 mm), leaflets coaptation ≥0.5 cm and systolic MV area of >2 cm^2^ were considered as criteria of successful repair.^20^, ^26^ (Further details are described in Data S1, references S51–52.)

Perioperative management is described in Data S2, references S53–55.

### Echocardiographic Measurements and Calculations

Resting and ESE were performed using commercially available ultrasound machines (Vivid 7 imaging device, GE Healthcare). All echocardiographic and Doppler data were obtained at rest and at peak exercise in digital format and stored on disks for offline analysis (EchoPAC, GE Vingmed Ultrasound AS, Horten, Norway). The Doppler tracings were averaged over 3 consecutive cardiac cycles for patients in sinus rhythm and 10 consecutive cycles for patients with atrial fibrillation.

MR was defined based on integrative criteria.^20^, ^27^ Severe MR was defined as effective regurgitant orifice area of ≥ 0.4 cm^2^; vena contracta ≥7 mm; regurgitant volume ≥60 mL; regurgitant fraction ≥50%.^27^ Recurrent MR was defined as a vena contracta width ≥3 mm at FU appointments in patients with no persistent MR at discharge.^18^, ^28^

Examination of the right ventricle was performed from a modified apical 4‐chamber view focused on the right ventricle. Tricuspid annular plane systolic excursion (TAPSE) was measured by M‐mode at the maximum displacement of the lateral tricuspid annulus. The RV fractional area change was calculated as follows: RV (end‐diastolic area‐end systolic area)×100% divided by RV end‐diastolic area. PA systolic pressure (PASP) was calculated by measuring the SP gradient between the right ventricle and right atrium, with the maximum velocity of the tricuspid regurgitation (TR) jet using the modified Bernoulli equation and estimated right atrial pressure.^29^ The TAPSE/PASP and the RV fractional area change/PASP ratios were taken as noninvasive indices of right ventricle to pulmonary circulation coupling.^30^

Complete details are described in Data S3; references S56–57.

## STATISTICAL ANALYSIS

The statistical analysis was conducted with R version 3.6.0 (2019‐04‐26, R Core Team 2019). R: A language and environment for statistical computing. R Foundation for Statistical Computing, Vienna, Austria. URL https://www.R‐project.org/) and STATA version 17.1 SE (Stata Corp LP, College Station, TX). Data were collected and tabulated with Microsoft Excel (Microsoft Corp). Continuous data are reported as mean±SD or as median with corresponding interquartile range (IQR). Discrete variables are reported as counts and percentages.

Normality distribution was assessed using Lilliefors (Kolmogorov–Smirnov) test. Inferential statistic between numerical variables (2 groups comparison) was conducted using Student's *t* test when normally distributed or Mann–Whitney test when not normally distributed. Comparative statistics between categorical variables was conducted with Pearson's chi‐square test. The comparison between repeated measures was conducted using ANOVA for repeated measures: multiple pairwise paired *t* tests between the levels of the within‐subjects factor (time point) were done adjusted using the Bonferroni multiple testing correction method.

Linear mixed models with random intercepts and random slope were conducted to identify independent predictors of 6‐MWT change at different timewaves of the FU, using restricted maximum likelihood estimation. Selection of significant predictors was performed using a mixed (backward and then forward) strategy, after controlling for potential confounders (such as age, gender, type of surgical intervention, residual MR).

Linear models were used also to evaluate the correlation between functional capacity and the main resting and exercise echocardiographic parameters. Pearson or Spearman correlation tests were used according to the distribution of the variables. All tests were 2 tailed and alpha level was set at 0.05 for significance. When a statistically significant relationship was found between exercise TAPSE/PASP and long‐term 6‐MWT, a receiver operating characteristic curve analysis was performed, in order to identify a TAPSE/PASP cutoff discriminating the lowest 6‐MWT quartile.^31^

### Power Analysis Calculation

By using 6‐MWT, as surrogate primary end point, measured at each FU evaluation in a longitudinal design, power was computed using 1‐way ANOVA for repeated measures.

Assuming a balanced study design and the sphericity assumption, we estimated to enroll n=192 patients to detect an effect size=0.3 with a power=0.80 and α=0.05.

### Intraobserver and Interobserver Variability Assessment

To examine intraobserver variability (repeatability), a random sample of N=5 (ie, 10%) of the overall echocardiographic examinations, performed at rest and at the preoperative baseline, was randomly selected for masked review by the same investigator (A.I.). To examine interobserver variability, a coinvestigator (A.P.), blinded to the clinical information and to the results of the first investigator, examined the other 5 randomly selected resting echocardiographic exams performed at the preoperative baseline. Intraclass correlation coefficients for the same observer and different observers were calculated by using previously described formulas^32^ for both TAPSE and PASP. The intraclass correlation coefficient for intrareader reproducibility was slightly greater for PASP (0.95 [95% CI, 0.87–1.03]) as compared with TAPSE (0.93 [95% CI, 0.80–0.1.05]). The intraclass correlation coefficient for interreader reproducibility was again higher for PASP (0.97 [95% CI, 0.92–1.02]) than for TAPSE (0.86 [95% CI, 0.63–1.09]).

## RESULTS

### Preoperative and Operative Data

The median preoperative 6‐MWT was 209 m (IQR, 39.5). Atrial fibrillation was present in 15 (30%) of the patients. The average anastomoses/patient was 2.2±1.2. An ablation of the atrial fibrillation was performed in 6 (12%) patients.

A restrictive mitral annuloplasty was performed using Carpentier‐Edwards Physio Ring (Edwards LifeSciencs), size 28 mm in 7 patients (14%), 30 mm in 9 patients (18%), and 32 mm in 4 patients (8%). In the valve replacement group, a Carpentier‐Edwards Perimount pericardial bioprosthesis (Edwards LifeSciences), 27 mm size was implanted in 9 (30%) patients, a 29 mm size in 9 (30%) patients, and a 31 mmm size in 6 (20%) patients. A mechanical St Jude Medical valve (St Jude Medical Inc, St Paul, MN) 27 mm size was implanted in 2 (7%), a 29 mm in 2 (7%), and a 31 mm (7%) in 2 patients. A tricuspid repair was performed in 4 (8%) patients. Complete data are shown in Table 1.

**Table 1:** Preoperative and Operative Data (Study Population=50 Patients)

Demographics	
Age, y	67.26±8.36
Female sex, n (%)	17 (34)
Body surface area, m^2^	1.85±0.17
Society of Thoracic Surgeons score	8.78±1.40
Clinical data	
Diabetes type II, n (%)	4 (8)
Hypertension, n (%)	34 (68)
Dyslipidemia, n (%)	20 (40)
Smoking history, n (%)	18 (36)
Cerebrovascular disease, n (%)	4 (8)
Preoperative intra‐aortic balloon pump, n (%)	8 (16)
AF, n (%)	15 (30)
Chronic kidney disease, n (%)	2 (4)
Functional	
New York Heart Association class III/IV, n (%)	13 (26)
6‐minute walking test distance, m	209 (39.5)
Medical therapy	
Angiotensin‐converting enzyme inhibitors, n (%)	48 (96)
Beta blocker, n (%)	48 (96)
Diuretics, n (%)	50 (100)
Nitrates, n (%)	36 (72)
Number of diseased vessels	
1, n (%)	11 (22)
2, n (%)	18 (36)
≥3, n (%)	21 (42)
Left anterior descending territory, n (%)	40 (80)
Circumflex territory, n (%)	17 (34)
RCA territory, n (%)	19 (38)
Surgery	
Cardiopulmonary bypass time, min	156.7±40.6
Aortic cross clamp time, min	113.6± 30.4
Coronary artery bypass grafting	
Anastomoses/patient	2.2±1.2
Arterial graft/patient	1.08±0.66
RCA+circumflex artery revascularization, n (%)	39 (78)
Conduits	
Left internal mammary artery, n (%)	42 (84)
Right internal mammary artery, n (%)	7 (14)
Radial artery, n (%)	5 (10)
Saphenous vein, n (%)	28 (56)
Prosthesis size	
27 mm, n (%)	11 (22)
29 mm, n (%)	11 (22)
31 mm, n (%)	8 (16)
Ring size	
28 mm, n (%)	7 (14)
30 mm, n (%)	9 (18)
32 mm, n (%)	4 (8)
Associated procedures	
Tricuspid repair, n (%)	4 (8)
AF ablation, n (%)	6 (12)

Continuous data are reported as mean±SD or as median with corresponding interquartile range. Discrete variables are reported as count and percentages. AF indicates atrial fibrillation; and RCA, right anterior descending.

### Resting and ESE Data

The median FU (ie, time from surgery to the last 6‐MWT and ESE assessment) was 6.5 years; IQR 3.70 (range: 4.5–8 years). At rest, the TAPSE significantly worsened from preoperative to long‐term FU (*P*=0.04), whereas the TAPSE/PASP ratio improved from 1 year to last FU (*P*<0.01). (Complete resting echo data are shown in Table 2.)

**Table 2:** Resting Echocardiographic Data at Baseline, at 1 Year and at the Last FU

	Preoperative n=50	1 y n=43	Last FU n=39	*P* value
Heart rate, bpm	74.2±3.0	74±3.6	75.1±3.2	0.25
Systolic blood pressure, mm Hg	134±3.7	133±3.9	132±4.6	0.11
LV end‐diastolic diameter, mm	62.6±3.5	59±6.35*	59.5±7.4†	<0.01
LV end‐systolic diameter, mm	51.7±2.0	47.7±5.4*	48.2±5.69	<0.01
LA diameter, mm	49.9±5.3	46.7±6.4*	47.2±7.3†	<0.01
LA volume, mL	72.3±12.4	67.4±9.8*	68.2±10.5†	<0.01
LV ejection fraction, %	35.4±4.9	37.2±4.6	35.9±7.05	0.22
LV end‐diastolic volume, mL	179.4±18.9	164.1±24.2*	165±25.1†	<0.01
LV end‐systolic volume, mL	150±8.5	128.9±24*	129.8±24.7† ^,^ ‡	<0.01
Stroke volume, mL	53.7±1.7	54.3±3.7	53.2±4.5‡	0.01
Cardiac output, L/min	4.3±0.3	4.7±0.5*	4.7±0.6† ^,^ ‡	<0.01
PM separation, cm	3.4±0.3	3.2±0.3*	3.10±0.4‡	<0.01
APM posterior displacement, cm	2.44±0.17	2.43±0.23	2.47±0.31‡	<0.01
APM lateral displacement, cm	1.81±0.12	1.95±0.17*	1.98±0.19†	<0.01
Posterior PM posterior displacement, cm	2.89±0.16	2.85±0.32	2.84±0.42‡	<0.01
Vena contracta width, mm	7±0.11	1.2±2.4	1.6±2.9	<0.001
Effective regurgitant orifice area, mm^2^	37.3±2.2	7.7±11*	13.3±18.4† ^,^ ‡	<0.01
Mitral regurgitant fraction, %	51.28±2.50	21.9±15.7*	15.04±21.2† ^,^ ‡	<0.01
Mitral regurgitant volume, mL	54.9±2.2	9.02±14.36*	16.33±23.43† ^,^ ‡	<0.01
PASP, mm Hg	40.98±5.59	39.09±6.33*	38.89±9.81‡	<0.01
Mitral peak gradient, mm Hg	3±0.3	8.1±0.65*	8.8±1.2† ^,^ ‡	<0.01
Mitral mean gradient, mm Hg	1.6±0.3	4.15±0.6*	4.4±0.7† ^,^ ‡	<0.01
Indexed effective orifice area, cm^2^/m^2^	…	1.34±0.29	1.32±0.30‡	<0.01
TAPSE, mm	17±3	16±5*	16±6†	0.04
TAPSE/PASP	0.43±0.09	0.44±0.19	0.47±0.27†	
RV FAC, %	32.36±7.63	32.64±11.5	34.3±13.87	0.23
RV FAC/PASP	0.80±0.22	0.89±0.41	1.02±0.57	0.04
Moderate TR, n (%)	4 (8)	0 (0)	1 (2)	0.02
Severe TR, n (%)	0 (0)	0 (0)	8 (16)	<0.01

Continuous data are reported as mean±SD or as median with corresponding interquartile range. Discrete variables are reported as count and percentages. *P* value is the overall value, overall significance of the 1‐way ANOVA for repeated measures model. APM indicates anterior papillary muscle; FU, follow‐up; LA, left atrium; LV, left ventricular; PASP, pulmonary arterial systolic pressure; PM, papillary muscle; RVFAC, right ventricular fractional area change; SBP, TAPSE, tricuspid annular plane systolic excursion; and TR, tricuspid regurgitation.

* Significant difference between preoperative and 1‐y postoperative.

^†^ Significant difference between preoperative and last follow‐up.

^‡^ Significant difference between 1‐y pos‐operative and last follow‐up.

Table 3 reports exercise echocardiographic data over time. Similarly to rest, the exercise TAPSE worsened from preoperative to long‐term FU (*P*<0.01), whereas TAPSE/PASP improved over time (*P*<0.01) (Table 3).

**Table 3:** Exercise Echocardiographic Data at Preoperative Baseline, at 1 Year and at the Last FU

	Preoperative n=50	1‐y n=43	Last FU n=39	*P* value
Workload, W	92.2±2.6	91.1±2.1*	91.2±2.4	0.16
Heart rate, bpm	121.22±4.26	122.6±6.4	122.4±5.5	0.21
Systolic blood pressure, mm Hg	175.02±5.15	174.8±5.4	175.4±4.9	0.75
LV end‐diastolic diameter, mm	59.5±3	54.9±8.9*	56.1±10† ^,^ ‡	<0.01
LV end‐systolic diameter, mm	49.6±2.2	46.7±6.2*	47.2±6.5	<0.01
LA diameter, mm	47.3±5.3	44.8±7.2*	45.9±8.3‡	0.02
LA volume, mL	69±10.4	65.5±9.6*	66.9±10.7‡	<0.01
LV ejection fraction, %	38.80±4.3	40.5±6.6	38±10.5	0.16
LV end‐diastolic volume, mL	171.3±17.7	160.8±28.3	161.9±28.6‡	0.02
LV end‐systolic volume, mL	144.3±8.7	123.3±27*	124.1±28.3‡	<0.01
Stroke volume, mL	61.5±2	62.3±9	59.8±10.6‡	0.14
Cardiac output, L/min	4.7±0.3	5.88±1.52*	5.8±1.6† ^,^ ‡	<0.01
PM separation, cm	3.2±0.2	3.1±0.4	3.0±0.6	0.12
APM posterior displacement, cm	2.36±0.23	2.43±0.39	2.51±0.47‡	0.04
APM lateral displacement, cm	1.95±0.08	2.01±0.23*	2.05±0.25‡	0.04
Posterior PM posterior displacement, cm	3.12±0.12	2.83±0.51*	2.83±0.59†	<0.01
Vena contracta width, mm	7.3±0.6	1.3±2.5*	1.7±3.1†	<0.01
Effective regurgitant orifice area, mm^2^	43.2±3.9	8.9±12.6*	14.8±20.7† ^,^ ‡	<0.01
Mitral regurgitant fraction, %	56.12±3.63	10.76±16.61*	17.37±25.44† ^,^ ‡	<0.01
Mitral regurgitant volume, mL	61.14±2.37	9.87±15.92*	17.89±25.77† ^,^ ‡	<0.01
PASP, mm Hg	48.56±7.91	44.65±9.45*	45.04±13.71†	<0.01
Mitral peak gradient, mm Hg	14.96±0.63	16.15±3.01*	17.00±3.59† ^,^ ‡	<0.01
Mitral mean gradient, mm Hg	7.4±0.4	9.3±1.8*	9.9±2.4† ^,^ ‡	<0.01
Indexed effective orifice area, cm^2^/m^2^	…	1.39±0.32	1.32±0.30‡	<0.01
TAPSE, mm	16.2±4.1	16±5.2	14.1±7.3† ^,^ ‡	<0.01
TAPSE/PASP ratio	0.35±0.12	0.39±0.18*	0.38±0.25†	<0.01
RVFAC, %	29.28±7.21	29.82±10.11	30.89±12.89	0.46
RVFAC/PASP	0.63±0.22	0.73±0.34	0.83±0.49	0.04
Severe tricuspid regurgitation, n (%)	0 (0)	0 (0)	16 (32)	<0.01

Continuous data are reported as mean±SD or as median with corresponding interquartile range. Discrete variables are reported as count and percentages. *P* value is the overall value, overall significance of the 1‐way ANOVA for repeated measures model. APM indicates anterior papillary muscle; FU, follow‐up; LA, left atrium; LV, left ventricular;PASP, pulmonary arterial systolic pressure; PM, papillary muscle; RVFAC, right ventricular fractional area change; and TAPSE, tricuspid annular plane systolic excursion.

* Significant difference between preoperative and 1‐y postoperative.

^†^ Significant difference between preoperative and last follow‐up.

^‡^ Significant difference between 1‐y postoperative and last follow‐up.

### Functional Capacity Data

The median preoperative 6‐MWT was 209 m (IQR, 39.5) at baseline; 240 m (IQR, 138.25) at 1 year and 255 m (IQR, 222.5) at long term.

**Figure 2:**
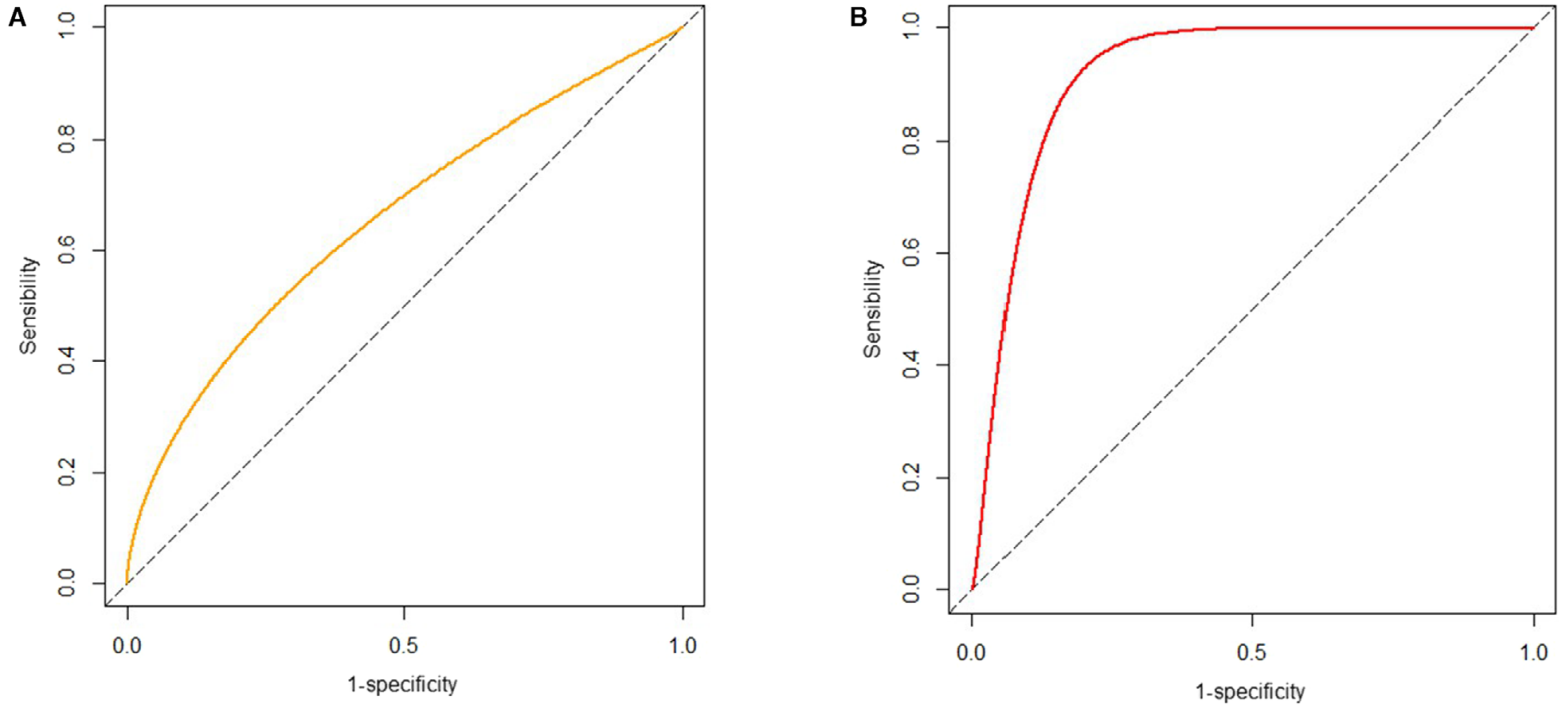
ROC curve. ROC depicting the sensitivity and the specificity of preoperative resting (**A**) and exercise (**B**) TAPSE/PASP in predicting the lowest 6‐MWT quartile. 6‐MWT indicates 6‐minute walking test, expressed in meters; PASP, pulmonary artery systolic pressure; ROC, receiver operating characteristic; and TAPSE, tricuspid annular plane systolic excursion.

The resting and exercise correlation between echocardiographic data and 6‐MWT is shown in Table 4. According to the receiver operating characteristic (analysis (Figure 2) a preoperative exercise TAPSE/PASP cutoff <0.34 predicted the lowest quartile of 6‐MWT at last FU (area under the curve: 0.92; sensitivity: 79%; specificity: 100%) and a composite clinical outcome of acutely decompensated heart failure and death for any cause (positive predictive value 91.3%, negative predictive value 100%).

**Table 4:** Correlation Between Preoperative Resting and Exercise Stress Echocardiographic Data and 6‐MWT Distance at 1 Year and at the Last FU

Echocardiographic data	6‐MWT at 1 y		6‐MWT at last FU	
	r	*P* value	r	*P* value
Resting				
LVEDD, mm	−0.21	0.16	−0.18	0.24
LVESD, mm	−0.1	0.51	−0.11	0.47
PASP, mm Hg	−0.05	0.73	−0.09	0.56
LVEF, %	0.03	0.72	0.02	0.8
EROA, mm^2^	−0.07	0.48	−0.07	0.54
Mitral RF, %	−0.15	0.18	−0.03	0.78
Mitral RV, mL	0.3	<0.01	0.38	<0.01
MMG, mm Hg	0.23	0.17	0.21	0.17
PASP, mm Hg	−0.05	0.73	−0.09	0.56
TAPSE, mm	0.5	<0.01	0.49	<0.01
TAPSE/PASP ratio	0.45	<0.01	0.51	<0.01
RVFAC, %	0.5	<0.01	0.53	<0.01
Exercise				
LVEDD, mm	−0.35	0.02	−0.3	0.04
LVESD, mm	−0.02	0.89	−0.03	0.81
LVEF, %	0.08	0.61	0.08	0.6
LV end‐diastolic volume, mL	0.09	0.55	0.02	0.87
LV end‐systolic volume, mL	−0.03	0.86	0.05	0.76
Cardiac output, L/min	0.04	0.78	0.03	0.83
EROA, mm^2^	−0.3	0.04	−0.43	<0.01
Mitral RF, %	−0.22	0.15	−0.18	0.24
Mitral RV, mL	0.15	0.33	0.15	0.33
PASP, mm Hg	−0.61	<0.01	−0.69	<0.01
MMG, mm Hg	−0.21	0.15	−0.22	0.13
TAPSE, mm	0.76	<0.01	0.74	<0.01
TAPSE/PASP ratio	0.77	<0.01	0.81	<0.01
RVFAC, %	0.49	<0.01	0.53	<0.01

6‐MWT indicates 6‐minute walking test; EROA, effective regurgitant orifice area; FU, follow‐up; LV, left ventricular; LVEDD, left ventricular end‐diastolic diameter; LVEDV, left ventricular end‐diastolic volume; LVEF, left ventricular ejection fraction; MMG, mitral mean gradient; PASP, pulmonary arterial systolic pressure; r, correlation test (Pearson correlation); RF, regurgitant fraction; RV, regurgitant volume; RVFAC, right ventricular fractional area; and TAPSE, tricuspid annular plane systolic excursion.

### Echocardiographic Predictors of 6‐MWT

Resting preoperative echocardiographic predictors of 6‐MWT, at late FU, are shown in Table S1. During exercise preoperative RV fractional area change, TAPSE, and TAPSE/PASP ratio were significantly associated with better long‐term 6‐MWT at univariable and multivariable analysis. Complete data are shown in Table 5.

**Table 5:** Exercise Preoperative Echocardiographic Predictors of 6‐MWT Distance at the Last FU

	Univariable					Multivariable				
	Estimates	SE	95% lower CI	95% upper CI	*P* value	Estimates	SE	95% lower CI	95% upper CI	*P* value
LA diameter, mm	1.03	0.71	−0.35	2.42	0.14					
LA volume, mm	0.33	0.36	−0.39	1.05	0.36					
APM lateral displacement, cm	31.76	46.04	−58.48	122	0.49					
APM posterior displacement, cm	10.22	16.08	−21.3	41.75	0.53					
Cardiac output, L/min	14.48	11.2	14.48	37.01	0.2					
LV ejection fraction, %	1.26	0.86	−0.47	2.99	0.15					
LV end‐diastolic diameter, mm	−1.44	1.28	−4.03	1.15	0.27					
LV end‐systolic diameter, mm	−0.29	1.77	−3.84	3.26	0.87					
LV end‐diastolic volume, mL	0.05	0.21	−0.38	0.48	0.82					
LV end‐systolic volume, mL	−0.006	0.43	−0.088	0.87	0.98					
Stroke, mL	3	1.84	−0.7	6.7	0.11					
Mitral mean gradient, mm Hg	8.7	8.9	−9.19	26.59	0.33					
Mitral peak gradient, mm Hg	−4.28	5.96	−16.26	7.7	0.48					
PM separation, cm	−10.53	20.68	−52.1	31.05	0.61					
PPM lateral displacement, cm	−5.21	41.97	−89.59	−5.21	0.9					
PPM posterior displacement, cm	−17.53	31.61	−81.09	46.03	0.58					
Mitral regurgitant volume, mL	−1.17	1.6	−4.38	2.04	0.47					
Effective regurgitant orifice area, mm^2^	1.31	0.98	−0.65	3.27	0.19					
Mitral regurgitant fraction, %	0.48	1.05	−1.62	2.58	0.65					
RVFAC, %	1.43	0.5	0.43	2.43	<0.01	1.13	0.52	0.11	2.15	0.03
PASP, mm Hg	0.42	0.48	−0.55	1.39	0.39					
TAPSE, mm	4.74	0.8	3.12	6.35	<0.01	4.05	0.9	2.24	5.87	<0.01
TAPSE/PASP	116.19	28.4	59.07	173.3	<0.01	106.91	31.54	45.09	168.73	<0.01

6‐MWT indicates 6‐minute walking test; APM, anterior papillary muscle; CO, cardiac output; EROA, FU, follow‐up; LA, left atrium; PASP, pulmonary artery systolic pressure; PM, papillary muscle; PPM, posterior papillary muscle; RF, regurgitant fraction; RV, regurgitant volume; RVFAC, right ventricle fractional area change; and TAPSE, tricuspid annular plane systolic excursion.

### Subgroup Analysis

The analysis of TAPSE/PASP and 6‐MWT according to the type of surgery is described in the supplemental results (Data S4) and in Figure S1 (Panel A and B) and Figure S2 (Panel A and B).

In the RMA group, 12 (n=12, 60%) patients developed MR recurrence over time, whereas in the MVR none of the patients showed recurrence of MR. In order to explore the association of this issue with TAPSE/PASP, 2 linear regression models were built (at 1 year and at last FU) only in patients with RMA (Data S4 and Table S2).

Furthermore, during exercise RMA group showed higher mean gradients over time (from preoperative to 1 year and from preoperative to last FU) (*P*<0.01 for both) (Table S3 and Figure S3).

### Clinical Outcomes

Clinical events were defined as a composite end point of acutely decompensated heart failure or death for any cause. During the FA 21 events were registered (42%). Survival analysis showed a survival rate of 92% at 1 year, 90% at 5 years, and 81.4% at 10 years.

**Figure 3:**
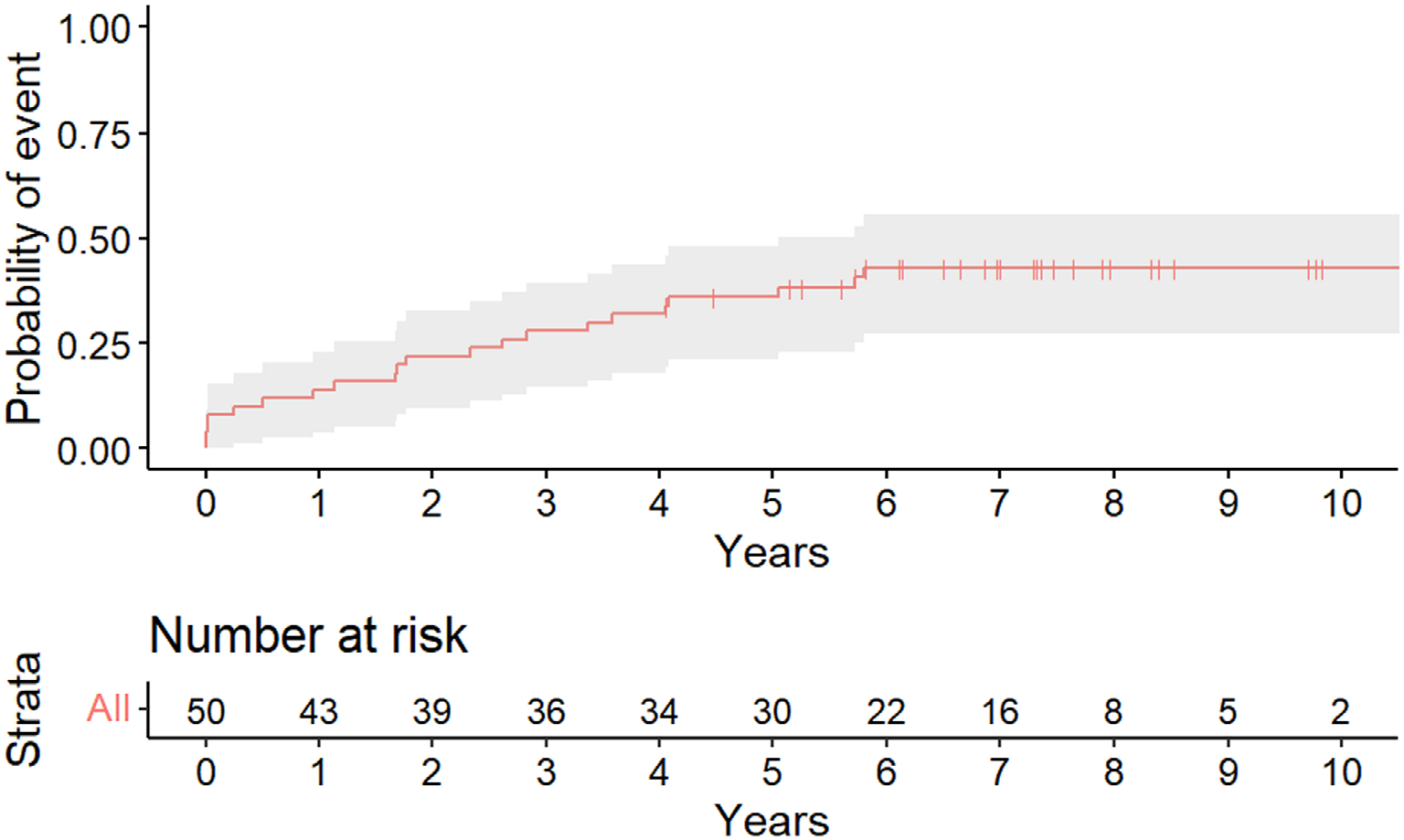
Kaplan–Meier curve for composite end point. Composite end point was defined as death for any cause or heart failure‐related hospitalization.

A preoperative exercise TAPSE/PASP <0.34 predicted the composite end point (*P*=0.01) (Figure 3).

A preoperative exercise TAPSE/PASP ratio <0.34 was not predictive of long‐term mortality (*P*=0.15) (Figure S4A). Conversely, the same variable emerged as a predictor of adverse events (ie, hospitalization for heart failure), *P*=0.01 (Figure S4B).

## DISCUSSION

The main findings of this study are as follows: (1) preoperative exercise TAPSE/PASP is strongly correlated to the 6‐MWT over time; (2) preoperative exercise TAPSE/PASP is an independent predictor of 6‐MWT at long term—a TAPSE/PASP ratio <0.34 predicts the lowest quartile of 6‐MWT; and (3) preoperative exercise TAPSE/PASP ratio has an excellent positive predictive value for clinical adverse events.

### Right Ventricular Function and Cardiac Surgery

The prognostic impact of the RV dysfunction after cardiac surgery is well known.^33^, ^34^ The underlying multifactorial mechanisms have been investigated in several studies.^35^ During cardiopulmonary bypass RV dysfunction may develop for a reduction of coronary flow to the interventricular septum, or for a suboptimal RV myocardial protection.^35^ We attempted to minimize the myocardial damage by using both antegrade and retrograde cardioplegia, the latter delivered into the vein grafting the right coronary artery.^36^ An incomplete revascularization may also play a role^35^ although we achieved a complete revascularization. The pericardiectomy may also results in an uncoupling between right ventricle and the pericardial sac.^33^ However, most of the studies on the right ventricle following cardiac surgery included mixed causes and procedures and lacked ESE data, making difficult to draw meaningful comparison.^33^, ^37^, ^38^

### The Role of RV‐Pulmonary Arterial Coupling

The so called right ventricular reserve, namely the capacity of the ventricle to increase contractility, may be more closely related to RV‐PA coupling than echocardiographic variables measured under resting conditions.^5^, ^6^ The RV‐PA coupling provide information on the RV function and its adaptation to increased afterload and potential damage.^4^ The gold‐standard assessment of the RV‐PA coupling that relies on pressure‐volume loops, by using conductance catheters,^6^ is a complex and demanding procedure. The TAPSE/PASP has therefore emerged as one of the few echocardiographic surrogates validated against pressure‐volume loops.^6^, ^7^ There is evidence about the prognostic impact of the RV‐PA coupling on outcome in several clinical settings.^7^, ^8^, ^10^, ^39^ Legris et al. found that TAPSE/PASP was a strong predictor of 6‐MWT.^9^ Likewise, Guazzi et al. observed that TAPSE/PASP ratio was a strong predictor of exercise tolerance.^10^ In another study the same author found a TAPSE/PASP cutoff ≥0.36 as better indicator of prognosis, when compared with isolated TAPSE and PASP.^4^ Tello et al. identified a TAPSE/PASP <0.31 as an indicator of worse prognosis in patients with pulmonary hypertension.^7^ Still Tello and colleagues described the concept of the RV‐PA reserve in detecting latent RV failure.^11^

The role of the RV‐PA coupling has been also investigated following transcatheter edge‐to‐edge repair.^39^, ^40^, ^41^, ^42^, ^43^ Trejo‐Velasco et al. found that a median TAPSE/PASP value ≤0.35 predicted clinical events and mortality.^40^ Karam et al. found that a TAPSE/PASP <0.274 predicted a worse outcome.^41^ A subanalysis of the COAPT (Cardiovascular Outcomes Assessment of the MitraClip Percutaneous Therapy for Heart Failure Patients With Functional Mitral Regurgitation) trial found RV‐PA uncoupling was associated with mortality and heart failure.^42^

In patients undergoing Mitra‐Clip procedures Adamo et al. found that TAPSE <15 mm, low postprocedural mean gradients, lack of cardiac surgery, and significant TR were predictors of TAPSE/PASP improvement.^39^ They identified a responder and a nonresponder group. In the nonresponder group the RV function and the TAPSE/PASP values were almost normal. They also found that, in the responder group, TAPSE improved with a PASP reduction.

Differently from Adamo et al., in our study the TAPSE worsened over time whereas TAPSE/PASP improved. The exercise improvement of RV‐PA coupling in our study is an intriguing finding for which there is no easy explanation. Following surgery most of the observed global left ventricular remodeling parameters improved; these findings along with the abolition of the MR, could explain the PASP reduction but not the worsened TAPSE. The improvement of TAPSE/PASP could be therefore related to a complex interplay between left and right ventricle or to other factors intrinsically related to the right ventricle.

In patients who underwent Mitra‐Clip also Shechter et al. found that a TASPE/PASP ≤0.34 was associated with adverse outcome.^43^ However, the mixed characteristics and lack of exercise data in most of the aforementioned studies differ from our report, making difficult to draw meaningful comparison.

In our study 16 (33%) patients developed a postoperative TR at last FU. In such a case, one could argue that TR may confound TAPSE measurement. Longitudinal parameters such as TAPSE, may be exaggerated in the setting of atrioventricular valve regurgitation^44^, ^45^

On the other hand, a study by Fortuni et al. investigated the TAPSE/PASP in 1149 patients with moderate‐to‐severe secondary TR. The TAPSE/PASP, as continuous variable, and a TAPSE/PASP <0.31 were added to a baseline model in order to evaluate their incremental prognostic value. The addition of TAPSE/PASP improved the predictivity of the model compared with the addition of standard echocardiographic parameters of RV systolic function (TAPSE or RV fractional area change). Furthermore, the addition of TAPSE/PASP <0.31 to the same model yielded a higher increase in predictivity compared with TAPSE <17 mm or RV fractional area change <35%.^46^

In patients treated with RMA, an exercise increment of the mean mitral gradients was observed over time, raising a doubt about a functional mitral stenosis with a potential impact on 6‐MWT. This concept was already reported by previous studies.^28^, ^47^

On the other hand, the aforementioned concept has been questioned by other studies that suggested other cardiac factors, rather than functional stenosis, could affect a patient's functional capacity.^48^, ^49^, ^50^

### Strengths and Limitations

This is the first prospective long‐term study to investigate the role of exercise RV‐PA coupling in predicting functional and clinical outcomes in patients undergoing surgery for SIMR. However, this study has several limitations. First, as a single‐center study, we cannot exclude the possibility of center‐specific bias. Second, it would be valuable to compare our findings with those from medical and noninvasive treatment arms. Additionally, we could not compare ESE data with cardiac magnetic resonance imaging and right heart catheterization.

Severe TR may confound TAPSE measurement. However other studies have investigated TAPSE/PASP in patients with TR and found that this parameter was superior to the conventional indices of RV systolic function. However, despite existing controversies, we acknowledge that in the presence of TR, our findings should be interpreted with caution.

We can assume, based on the exercise increment of the mean gradients in the RMA, a potential confounding factor affecting the interpretation of the results. This hypothesis remains to be determined with larger and more homogeneous studies. However, the correlation and the impact of a potential functional mitral stenosis on TAPSE/PASP ratio is beyond the scope of the current study.

Due to logistical reasons, we were unable to recruit the number of patients initially planned based on the statistical power analysis. Hence, the limited sample size may affect the interpretation of the results, potentially leading to a classic type II error. In this respect our findings should be considered as hypothesis generating and will need to be validated by larger studies.

## CONCLUSIONS

In patients with ischemic MR undergoing surgery, the preoperative exercise TAPSE/PASP ratio strongly correlated with the 6‐MWT distance. A preoperative exercise TAPSE/PASP <0.34 predicted a poor 6‐MWT performance and a higher likelihood of clinical adverse events over time. In such challenging patients the assessment of exercise RV‐PA coupling, paired with 6‐MWT, could be potentially useful to improve risk stratification. Larger studies are required to confirm these findings.

## Sources of Funding

The British Heart Foundation Cardiac Surgery Chair (CH/17/1/32804) has supported this work. P. Pibarot is the Canada Research Chair in Valvular Diseases funded by Canadian Institutes of Health Research (CIHR), Ottawa, Ontario, Canada. His research program is funded by a Foundation Grant (FDN‐143225) from CIHR.

## Disclosures

P. Pibarot received research grant funding from Cardiac Phoenix that develops the basal annuloplasty of the cardia externally device for the treatment of ischemic mitral regurgitation. The remaining authors have nothing to disclose.

## Supporting information

Data S1–S4
Tables S1–S3
Figures S1–S4
References 51–57
