# Supplementary material for: Exercise Right Ventricular‐Pulmonary Arterial Coupling and Functional Outcome in Patients Undergoing Surgery for Secondary Ischemic Mitral Regurgitation
Source: J Am Heart Assoc. 2025 Apr 11;14(8):e037198. doi: 10.1161/JAHA.124.037198 (PMC12132878; doi:10.1161/JAHA.124.037198)
Supplement: Supplementary file 1 [file JAH3-14-e037198-s001.pdf]

SUPPLEMENTAL MATERIAL

SUPPLEMENTAL METHODS

Data S1: Surgery

Tricuspid regurgitation 2+ or greater or dilated tricuspid annulus (more than 21 mm/m2, indexed to body surface area, BSA) were indications for tricuspid annuloplasty (51), which was accomplished with a Carpentier Edwards ring (Edwards Lifesciences, Irvine, CA, USA). The ring size was selected on the basis of the length of the tricuspid septal leaflet attachment and the surface area of the anterior leaflet, then downsized by at least 1 measure. Ring implantation was performed by placing 8-10 mattress sutures through the tricuspid annulus around the orifice, avoiding the area of atrioventricular conduction tissue (52).

The surgical revascularization (CABG) strategy was individually planned in each patients according to his state, with the main goal of no increase in mortality or morbidity. In single internal mammary artery (IMA) grafting procedure, patients received a single left IMA (LIMA) graft to the left anterior descending artery (LAD) plus supplemental vein grafts to other coronary arteries. In bilateral mammary artery (BIMA) grafting, patients received both left and right mammary arteries to the most important coronary territories on the left side: preferentially, LIMA to the LAD system and RIMA to the circumflex artery (CX) system, with supplemental vein grafts to other coronary territory. The arterial grafts (LIMA and/or RIMA) were used as in situ grafts as first choice and, when unfeasible, as composite grafts as second option. Sequential IMA graft were performed according to the coronary lesions.

Data S2: Perioperative management

Immediately after surgery, in patients who received mechanical prostheses anticoagulation was obtained by subcutaneous unfractionated heparin, bridged to warfarin up to therapeutic INR range (2.5 to 3.5). In patients with biological prostheses the above anticoagulation protocol was maintained only for 3 months.

In patients with atrial fibrillation or other risk factors for thromboembolic sequelae, the same was

maintained indefinitely. Additional antiplatelet agent was initiated and maintained in both groups (53). In all patients, an appropriate guidelines-directed medical therapy, including the use of statin and B- blockers therapy was instituted **(**54-55).

Data S3: Echocardiographic measurements

Left ventricular end-diastolic (LVED), end-systolic volumes (ESV) and LV ejection fraction (EF) were measured by using the modified Simpson's biplane method (21,56). The stroke volume (SV) and cardiac output (CO) measured as previously described (22).

Mitral valve tenting area was measured by the area enclosed between annular plane and mitral leaflets from the parasternal long-axis view at mid-systole (56). Coaptation height measured as by the distance between leaflet coaptation and the mitral annulus plane in the apical four chamber view (56). The distance between the posterior papillary muscle head and the intervalvular fibrosa (PPM fibrosa) in the long-axis view measured the apical displacement of the posterior papillary muscle (56). Lateral and posterior displacements of papillary muscles were evaluated from the short-axis view (56). Separation between papillary muscles was directly measured from body to body in the short-axis view (26). Peak, mean trans-mitral pressure gradients and mitral valve effective orifice area (EOA) were measured as described elsewhere (57).

SUPPLEMENTAL RESULTS

Table S1. Resting preoperative echocardiographic predictors of 6-MWT distance at late FU

	Estimates	SE	95% Lower CI	95% Upper CI	p
**LA diameter, mm**	0.81	0.7	-0.61	2.22	0.26
**LA volume, mm**	0.24	0.31	-0.38	0.24	0.85
**APM lateral displacement, cm**	-52.8	29.1	-111.5	5.94	0.08
**APM posterior displacement, cm**	31.8	20.8	-10.1	73.6	0.13
**CH, cm**	40.4	44.4	-49.1	129.9	0.40
**CO, L/min**	13.52	12.5	-11.7	38.7	0.28
**LVEF, %**	0.36	0.80	-1.26	1.98	0.66
**LVEDD, mm**	-0.98	1.05	-3.1	1.14	0.36
**LVESD, mm**	0.49	1.96	-3.46	4.44	0.80
**LVEDV, ml**	0.16	0.19	-0.22	0.54	0.40
**LVESV, ml**	0.14	0.45	-0.77	1.05	0.76
**SV, ml**	-0.67	2.21	-5.13	3.78	0.76
**MMG, mmHg**	5.27	13.17	-21.3	31.8	0.69
**MPG, mmHg**	-10.02	11.25	-32.7	12.66	0.38
**TA, cm2**	-6.31	10.79	-28.07	15.44	0.56
**PM separation, cm**	-15.44	14.36	-44.38	13.51	0.29
**PPM lateral displacement, cm**	-51.95	29.52	-111.44	7.54	0.09
**PPM posterior displacement, cm**	15.98	22.68	-29.73	61.70	0.48
**Mitral RV, ml**	-0.74	1.73	-4.22	2.73	0.67
**EROA, mm2**	2.74	1.64	-0.58	6.05	0.10
**Mitral RF, %**	0.48	1.05	-1.62	2.58	0.65
**RVFAC, %**	1.14	0.46	0.22	2.07	0.02
**PASP, mmHg**	0.97	0.70	-0.44	2.38	0.17
**TAPSE, mm**	2.27	1.32	-0.40	4.94	0.09
**TAPSE/PASP ratio**	37.14	40.11	-43.5	117.78	0.36

SE: standard error; CI: confidence interval.

LA: left atrium; APM: anterior papillary muscle; CH: coaptation height; CO: cardiac output; LVEF: left ventricular ejection fraction; LVEDD: left ventricular end diastolic diameter, LVESD: left ventricular end systolic volume; LVEDV: left ventricular end- diastolic volume; LVESV: left ventricular end-systolic volume; SV: stroke volume; MMG: mitral mean gradient; MPG: mitral peak gradient; TA: tenting area; RV:regurgitant volume; EOA: effective orifice area; RF: regurgitant fraction; RVFAC: right ventricular fractional area change; PASP: pulmonary artery systolic pressure; TAPSE: tricuspid annular plane systolic excursion; FU: follow-up

Data S4: Subgroup Analysis

Six -MWT and TAPSE/PASP ratio according to the type of surgery (MVR vs RMA)

Mitral valve replacement (MVR) was associated with improvement in 6-MWT distance over time, whereas the RMA showed a deterioration (*Figure S1, A and B*). The resting and exercise TAPSE/PASP ratio improved in MVR group, from preoperative baseline to the last FU, whereas it worsened in patients with RMA (*Figure S2, A and B*)

Figure S1 (A and B). 6-MWT distance over time. Analysis by surgery type (MVR vs. RMA)

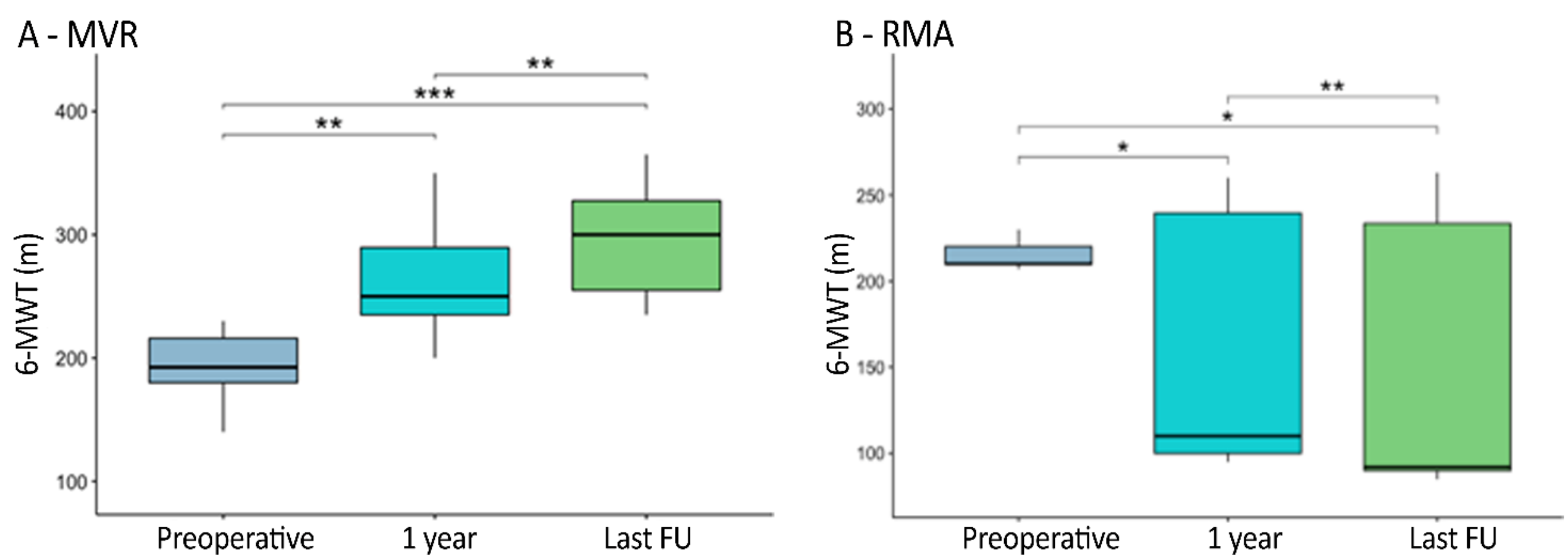

ANOVA repeated measure $p < 0.01$. ANOVA repeated measure $p = 0.003$ for overall model

* denotes significant differences at each timepoint

A significant difference of 6-MWT was found from preop to 1 year and from 1 year to last FU

6-MWT: six-minute walking test; m: meters; MVR: mitral valve replacement, RMA: restrictive mitral annuloplasty; FU: follow-up

Figure S2. Resting (A) and Exercise (B) TAPSE/PASP ratio over time. Analysis by surgery type (MVR vs RMA)

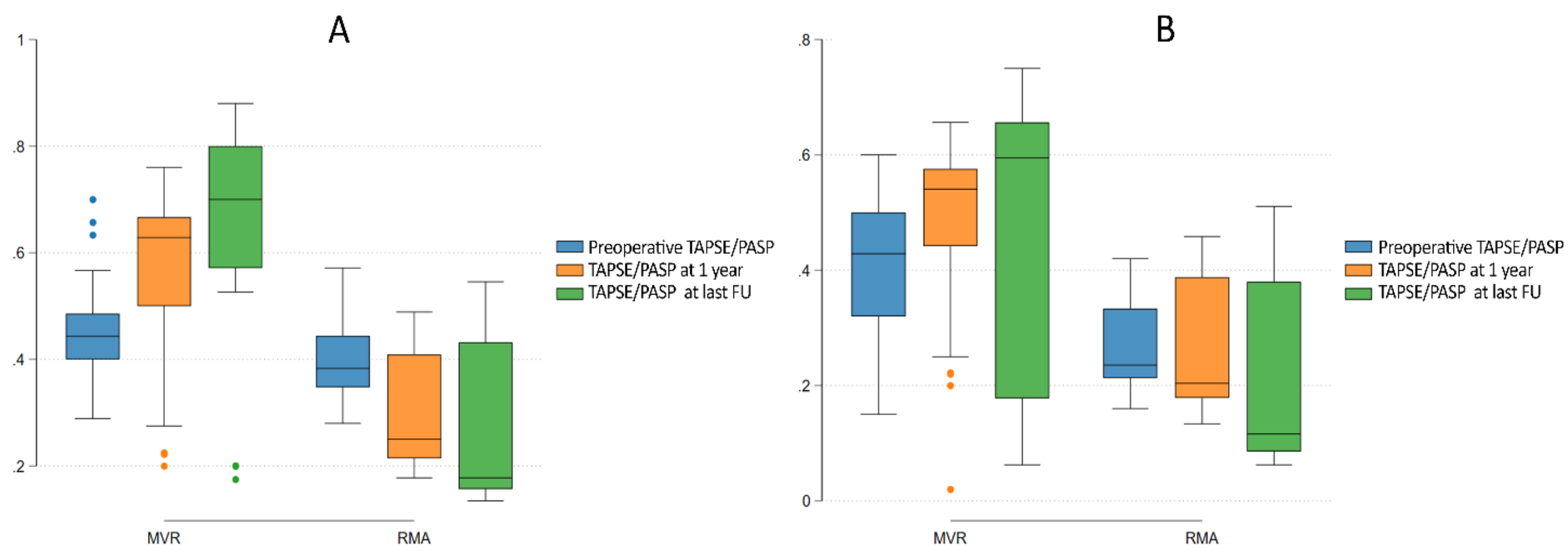

A. Two- way ANOVA for repeated measures, by surgery type: Overall Model p-value < 0.001; Surgery procedure performed, p < 0.001

Change of resting TAPSE/PASP over time, p = 0.60. Interaction between over time change in resting TAPSE/PASP and type of surgery, p < 0.001

B. Two-way ANOVA for repeated measures, by surgery type: overall Model p < 0.001.

Surgery procedure performed, p < 0.001. Change of exercise TAPSE/PASP over time, p = 0.46. Interaction between over time change in TAPSE/PASP and type of surgery, p = 0.002

MVR: mitral valve replacement; RMA: restrictive mitral annuloplasty; TAPSE: tricuspid plane systolic excursion; PASP: pulmonary artery systolic pressure; FU: follow-up.

TAPSE/PASP and MR recurrence

Twelve (n= 12, 60%) patients developed MR recurrence over time. In order to explore the association of this issue with TAPSE/PASP, two linear regression models were built (1 year and last follow-up). This analysis was done only in patients with restrictive mitral annuloplasty, because patients who had valve replacement did not have recurrence, and therefore they were not representative of the entire study population. This analysis showed an inverse correlation between

those two parameters.

A significant inverse relationship between MR recurrence and resting TAPSE/PASP ratio was found at 1 year ($p < 0.01$, $R2 = 0.76$, with a 0.18-unit decrease in TAPSE/PASP in case of MR recurrence), and at long-term follow-up ($p < 0.01$, $R2 = 0.76$, 0.25-unit decrease).

A significant inverse relationship between MR recurrence and exercise TAPSE/PASP ratio ($p < 0.01$, $R2 = 0.76$), with a 0.20-unit decrease in TAPSE/PASP in case of MR recurrence, was found at 1 year and at long-term follow-up ($p < 0.01$, $R2=0.76$, 0.28-unit decrease). (*Table S2*)

Table S2. Linear regression model. Prediction of TAPSE/PAPS on MR recurrence

**Resting Echocardiogram**	**Est.**	**SE**	**p**
MR recurrence vs. TAPSE/PASP at 1 year	-0.29	0.05	<0.01
MR recurrence vs. TAPSE/PASP at last FU	-0.27	0.05	<0.01
**Exercise Echocardiogram**	**Est.**	**SE**	**p**
MR recurrence vs. TAPSE/PASP at 1 year	-0.39	0.07	<0.01
MR recurrence vs. TAPSE/PASP at last FU	-0.36	0.07	<0.01

Linear regression model for predicting MR recurrence from resting and exercise TAPSE/PAPS
Analysis performed only in patients referred to RMA. Est: estimate; SE: standard error
MR: mitral regurgitation; TAPSE: tricuspid annular plane systolic excursion; PASP: pulmonary artery systolic pressure; RMA: restrictive mitral annuloplasty; FU: follow-up

Forward gradients in the RMA group

Table S3. Resting and exercise mean gradients in the RMA group, at each time point.

**Resting Echo**	**Pre-operative**	**1-Year**	**Last FU**	**p-value**
**MMG, mmHg**	1.5 (1.5-2.0)	4.5 (4.4-4.7) †	4.8 (4.7-5.2) § #	< 0.01
**Exercise Echo**	**Pre-operative**	**1-Year**	**Last FU**	**p-value**
**MMG, mmHg**	7.7 (7.2-7.8)	10.8 (10-12.2) †	12.6 (10.2-13.4) §	< 0.01

One-way ANOVA for repeated measures model

Continuous data are reported as median and IQR. FU: follow-up; RMA: restrictive mitral annuloplasty; MMG: mitral mean gradient.

†: significant difference between preoperative and 1-year post-operative

§: significant difference between preoperative and last follow-up

#: significant difference between 1-year post-operative and last follow-up

p-value = overall significance of the One-way ANOVA for repeated measures model

Figure S3. Box-plot depicting resting and exercise mean gradients in the RMA, at each time point

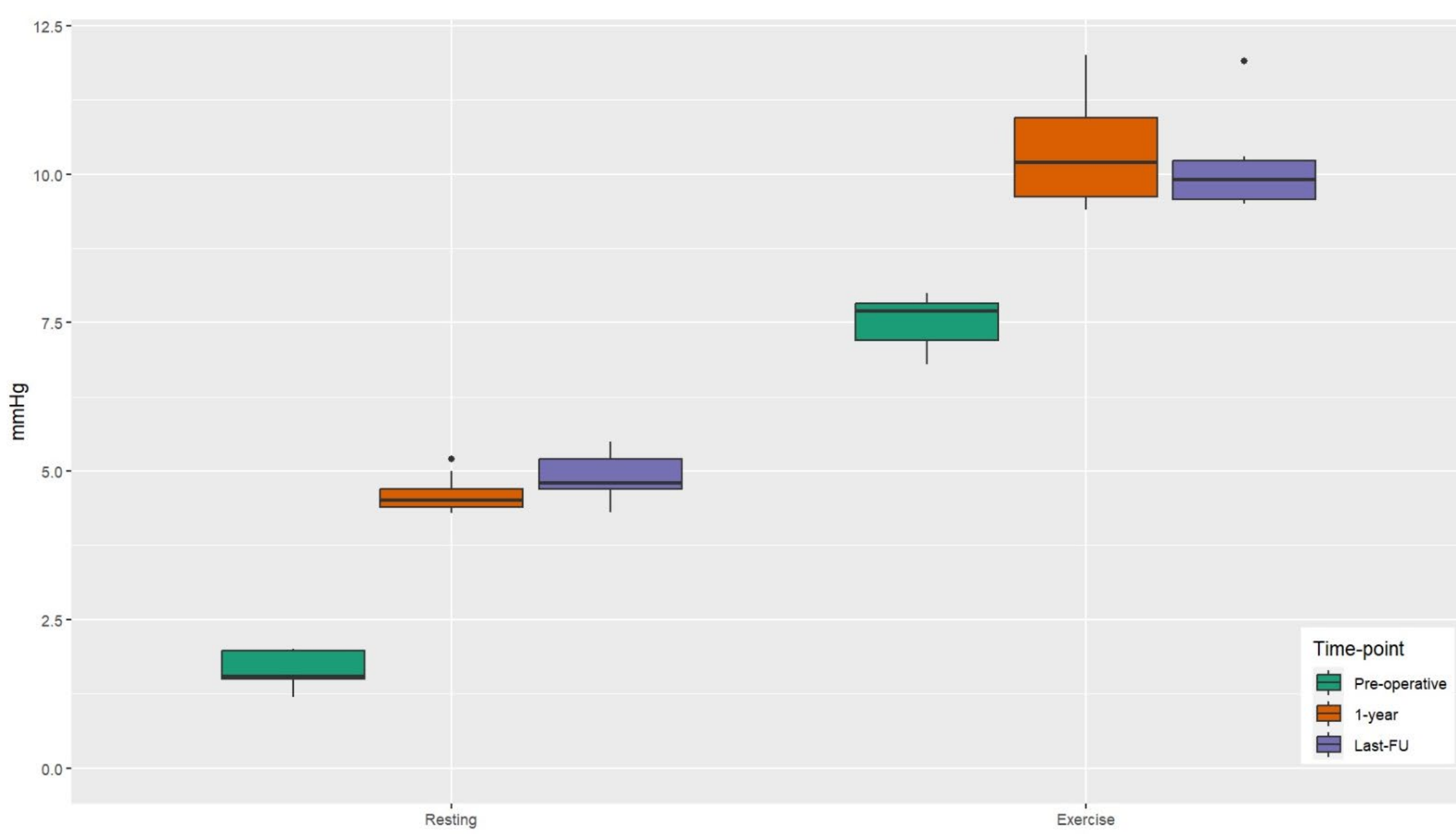

RMA: restrictive mitral annuloplasty; FU: follow-up

Preoperative RV-PA coupling and clinical outcomes.

Figure 4. (**A and B**)

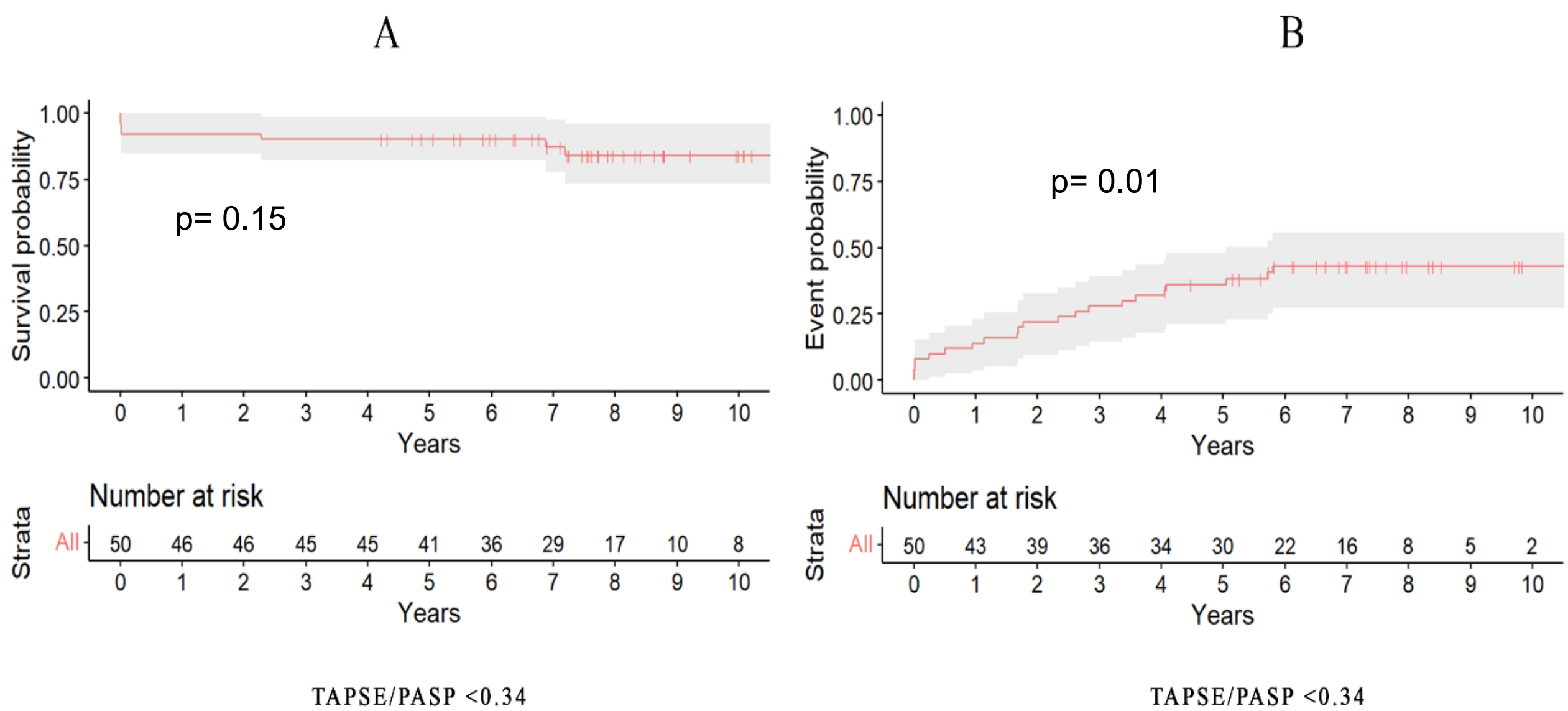

TAPSE: tricuspid annular plane systolic excursion; PASP: pulmonary artery systolic pressure
